# Case Report: Robot-assisted nissen fundoplication in spinal muscular atrophy type 2—a tailored surgical and anaesthetic strategy

**DOI:** 10.3389/fsurg.2026.1964655

**Published:** 2026-09-21

**Authors:** Michela Cing Yu Wong, Marco Agamennone, Francesca Di Domenicantonio, Girolamo Mattioli

**Affiliations:** 1 Department of Pediatric Surgery, IRCCS Istituto Giannina Gaslini, Genoa, Italy; 2 Department of Neuroscience, Rehabilitation, Ophthalmology, Genetics and Maternal Child Science, University of Genoa (DINOGMI), Genova, Italy

**Keywords:** case report, gastro esophageal reflex disease, nissen fundoplication, robotic surgery, SMA

## Abstract

Antireflux surgery may be required in patients with spinal muscular atrophy type 2 (SMA2), but disease-specific experience is limited. To our knowledge, we report the first robot-assisted Nissen fundoplication in SMA2. A 23-year-old woman with severe gastroesophageal reflux disease, bulbar dysfunction, impaired secretion management, severe restrictive respiratory impairment, a previous failed intubation, and fixed musculoskeletal deformities was referred for surgery. Multidisciplinary planning led to fibreoptic nasotracheal intubation under deep sevoflurane sedation and topical anaesthesia, preserving spontaneous ventilation. Intubation was achieved on the first attempt without desaturation or haemodynamic instability. Robot-assisted Nissen fundoplication with Mikulicz pyloroplasty was completed using adapted positioning and port placement. Robotic liver retraction and suction through a 10-Fr nasogastric tube introduced via a robotic port avoided additional access. Positioning required 30 min, operative time was 110 min, including 65 min of console time, and total procedural time from the start of positioning to skin closure was 140 min. After complete neuromuscular recovery, the patient was extubated 2 h 43 min after surgery directly onto non-invasive ventilation. No complications occurred, and she was discharged on postoperative day 5. At 1 month, contrast imaging confirmed the absence of reflux and satisfactory gastric emptying. At a 4-month multidisciplinary clinical assessment, she reported no reflux-related symptoms and improved management of salivary secretions during ongoing follow-up with our logopedists. Robot-assisted Nissen fundoplication in SMA2 is feasible in expert hands when supported by tailored anaesthetic and surgical planning.

## Introduction

Spinal muscular atrophy type 2 (SMA2) is characterized by early-onset muscle weakness, generalized hypotonia, and progressive motor impairment due to motor-neuron degeneration (1). It is increasingly recognized as a multisystem disorder with gastrointestinal involvement, including dysphagia, delayed gastric emptying, and gastroesophageal reflux disease (GERD) (2–4). In neuromuscular disease, GERD may further increase pharyngeal contamination and upper-airway secretion burden through regurgitation and local acid irritation of the oropharyngeal and posterior pharyngeal mucosa (5). Antireflux surgery has been associated with a reduced, although not eliminated, risk of aspiration pneumonia in neurologically impaired patients with GERD (6). Reflux may therefore represent a modifiable component of a multifactorial aspiration risk in selected patients (7).

Fundoplication in SMA has previously been performed laparoscopically, including in a small series of patients with type I or severe type II disease and in a recent paediatric SMA2 case (8, 9). The combined laparoscopic Nissen, gastrostomy, and NIV strategy was reported to optimize quality of life in this population (8). Robot-assisted Nissen fundoplication has also been described in neurologically impaired children with pre-existing gastrostomy (10). However, our updated literature search identified no report specifically combining robot-assisted fundoplication with SMA. The present report examines how robotic surgery was integrated with individualized anaesthetic planning in this high-risk setting.

### Case description

A 23-year-old woman (weight 30 kg, height 153 cm, body mass index 13 kg/m^2^) with SMA2 confirmed by SMN1 gene testing was referred for surgical treatment of severe GERD in the setting of impaired management of oropharyngeal secretions. She was classified as ASA III and was receiving risdiplam, with the last dose administered at 06:00 on the morning of surgery. Relevant comorbidities included severe scoliosis, with pre-fusion Cobb angles documented 45°, treated by spinal fusion in 2013; heterozygous Factor V Leiden mutation; severe bulbar dysfunction with dysphagia for liquids; and nocturnal non-invasive ventilation (NIV) for 9 h nightly. Her home respiratory equipment included an ASTRAL 150 ventilator in pressure-support mode (inspiratory pressure 8 cmH₂O, rise time 250 ms, inspiratory time 1.0–1.5 s) and a mechanical in-exsufflator (+40/−45 cmH₂O).

Table 1 presents a timeline of the patient's clinical history before surgery.

**Table 1:** Clinical timeline summarizing the patient's disease course.

Time point	Clinical event
6–8 months of age	Started crawling, although with some difficulty.
Approximately 1 year of age	Achieved standing only with support; independent ambulation was not acquired.
July 2003	Molecular diagnosis of spinal muscular atrophy type 2 (SMA2).
Childhood	A cough-assist device was introduced during primary-school years.
October 2013	Spinal fusion for severe scoliosis.
2014	Initiation of regular nocturnal non-invasive ventilation.
2016	Initiation of risdiplam within the SUNFISH clinical trial, without adverse effects.
February 2020	Pneumococcal pneumonia, successfully treated at home.
Summer 2022	Mild SARS-CoV-2 infection, with symptoms resolving after approximately 5 days.
February–March 2023	Progressive dysphagia, transition from solid to blended food, and approximately 10-kg weight loss.
May 2024	Radiologically inserted gastrostomy for severe malnutrition.
July 2024	Planned otorhinolaryngological procedure abandoned after failed direct and video-laryngoscopic intubation.
Preoperative assessment	Upper gastrointestinal contrast study demonstrated severe gastroesophageal reflux in the context of impaired secretion management.
April 2026	Robot-assisted Nissen fundoplication

### Clinical findings

Pulmonary function testing showed severe restriction: forced vital capacity 25% predicted, forced expiratory volume in 1 s 22% predicted, and an FEV₁/FVC ratio of 0.77. Arterial blood gas analysis on room air showed pH 7.47, PaO₂ 109 mmHg, PaCO₂ 36.9 mmHg, HCO₃⁻ 27 mEq/L, and SpO₂ 96%; SpO₂ during NIV was 98%. Airway assessment demonstrated Mallampati class 4, mouth opening below 5 mm, absent mandibular protrusion (upper-lip bite test class C), a fixed cervical spine with obligate right-lateral head position, and severe bulbar dysfunction. During a previously planned otorhinolaryngological procedure in 2024, both direct laryngoscopy and videolaryngoscopy had failed because tracheal access was anatomically impossible; that procedure was abandoned and the patient awakened without complication.

### Diagnostic assessment and preoperative planning

A preoperative upper gastrointestinal contrast study demonstrated marked gastroesophageal reflux. Although oesophageal pH-metry is the gold-standard test for the diagnosis of GERD, it was not performed in this patient. After collegial review of the radiological and clinical findings, gastroenterologists, physiatrists, logopedists, surgeons, and anaesthesiologists considered the documented marked reflux, persistent clinical burden, and overall complexity sufficient to establish the indication for antireflux surgery. Because swallowing dysfunction and ineffective cough already compromised airway protection and secretion clearance, GERD was considered an additional and potentially modifiable source of pharyngeal contamination and aspiration.

The indication for antireflux treatment and the available approaches were reviewed. Transoral incisionless fundoplication, an established endoscopic approach (11), was considered and discussed with the patient and her family. However, given the markedly restricted mouth opening and exceptionally high anaesthetic risk associated with prolonged transoral instrumentation, the multidisciplinary team did not consider this approach appropriate in this individual case. Furthermore, endoscopic fundoplication is not performed at our institution, whereas the surgical and anaesthetic teams have extensive experience with robot-assisted procedures in patients with complex neurological conditions. Following multidisciplinary evaluation, robot-assisted Nissen fundoplication was selected with the aim of achieving durable reflux control while minimising the likelihood of recurrence and subsequent reintervention. This was particularly important because any repeat procedure would expose the patient to the same exceptional airway and respiratory risks. The rationale, expected benefits, and potential risks were discussed with the patient, with her mother involved at the patient's request.

Concomitant Mikulicz pyloroplasty was also planned preoperatively because delayed gastric emptying is a recognised gastrointestinal manifestation of SMA (3, 4). In selected neurologically impaired patients, our team tends to include pyloroplasty to facilitate gastric emptying and reduce postoperative abdominal distension.

The documented failed intubation and severely restricted pulmonary reserve left little margin for apnoea or repeated airway instrumentation. Joint anaesthetic and surgical planning therefore identified fibreoptic nasotracheal intubation with preserved spontaneous ventilation as the definitive airway strategy. Awake fibreoptic intubation was considered; deep inhalational sedation was selected to improve tolerance of nasal instrumentation while maintaining spontaneous ventilation until the airway had been secured. Two anaesthesiologists were assigned to intubation, the intensive care unit (ICU) was notified in advance, and a difficult-airway trolley containing a hyperangulated videolaryngoscope, cricothyrotomy kit, and surgical tracheostomy set was kept immediately available. If fibreoptic intubation failed while spontaneous ventilation and oxygenation remained adequate, sedation would be discontinued and the patient awakened. Emergency front-of-neck access or surgical tracheostomy was reserved for failure of ventilation or oxygenation.

From the surgical perspective, joint contractures, previous spinal fusion, a narrow abdominal domain, and a pre-existing gastrostomy were expected to restrict positioning and instrument angulation. A tailored robot-assisted approach was planned to obtain upper-abdominal exposure while minimizing arm collisions and avoiding prolonged pneumoperitoneum.

## Therapeutic intervention

### Airway management and anaesthetic strategy

The patient entered the operating room in her wheelchair and was positioned semi-recumbent at 45°. Standard monitoring, peripheral venous access, and cerebral near-infrared spectroscopy were established before sedation. The nasal cavity and oropharynx were topicalised with 10% lidocaine, followed by right-nasal vasoconstrictor instillation and preoxygenation with 100% oxygen through an endoscopy mask. Deep inhalational sedation was induced with sevoflurane 6% in oxygen while spontaneous ventilation was preserved. After loss of verbal response and the eyelash reflex, additional laryngotracheal topicalisation was performed under fibreoptic vision using a spray-as-you-go technique with 2% lidocaine. The cumulative lidocaine dose, including all 10% and 2% lidocaine administered by both routes, was 60 mg (2.0 mg/kg), within recommended limits (12–15).

Nasotracheal intubation through the right nostril used a 3.7-mm paediatric fibrescope loaded with a 5.5-mm cuffed reinforced endotracheal tube. Tracheal intubation was achieved in one attempt in under 2 min without desaturation or haemodynamic instability, and tube position was confirmed by capnography and visualisation of the carina (16). A radial arterial line and central venous catheter were then placed under general anaesthesia. Anaesthesia was maintained with sevoflurane, remifentanil, and titrated rocuronium. Succinylcholine was avoided because of the risk associated with depolarising neuromuscular blockade in SMA, while non-depolarising blockade was monitored carefully (17, 18). Pressure-controlled ventilation was adjusted during pneumoperitoneum using 5 cmH₂O of positive end-expiratory pressure and by increasing respiratory rate and inspiratory pressure in response to rising end-tidal carbon dioxide (19). Oxygen saturation and haemodynamics remained stable, and no vasopressor support or transfusion was required. Analgesia was opioid-sparing and based on paracetamol and ibuprofen.

### Patient positioning and robotic setup

After airway securement, the patient was positioned supine with extensive pressure-point padding. Lower-limb contractures required progressive adjustment to obtain stable alignment without mechanical stress. This positioning phase required 30 min before trocar placement. The table was tilted approximately 20° in reverse Trendelenburg to improve access to the oesophageal hiatus (Figure 1). An 8-mm robotic trocar was inserted by an open supraumbilical technique, approximately 4 cm above the umbilicus, and pneumoperitoneum was established at 12 mmHg. Three additional 8-mm robotic trocars were placed approximately 5 cm apart along a high transverse supraumbilical line. The higher, more vertical working axis reduced external arm collisions while accommodating the pre-existing gastrostomy (Figure 2). The restricted abdominal domain precluded an accessory trocar.

**Figure 1:**
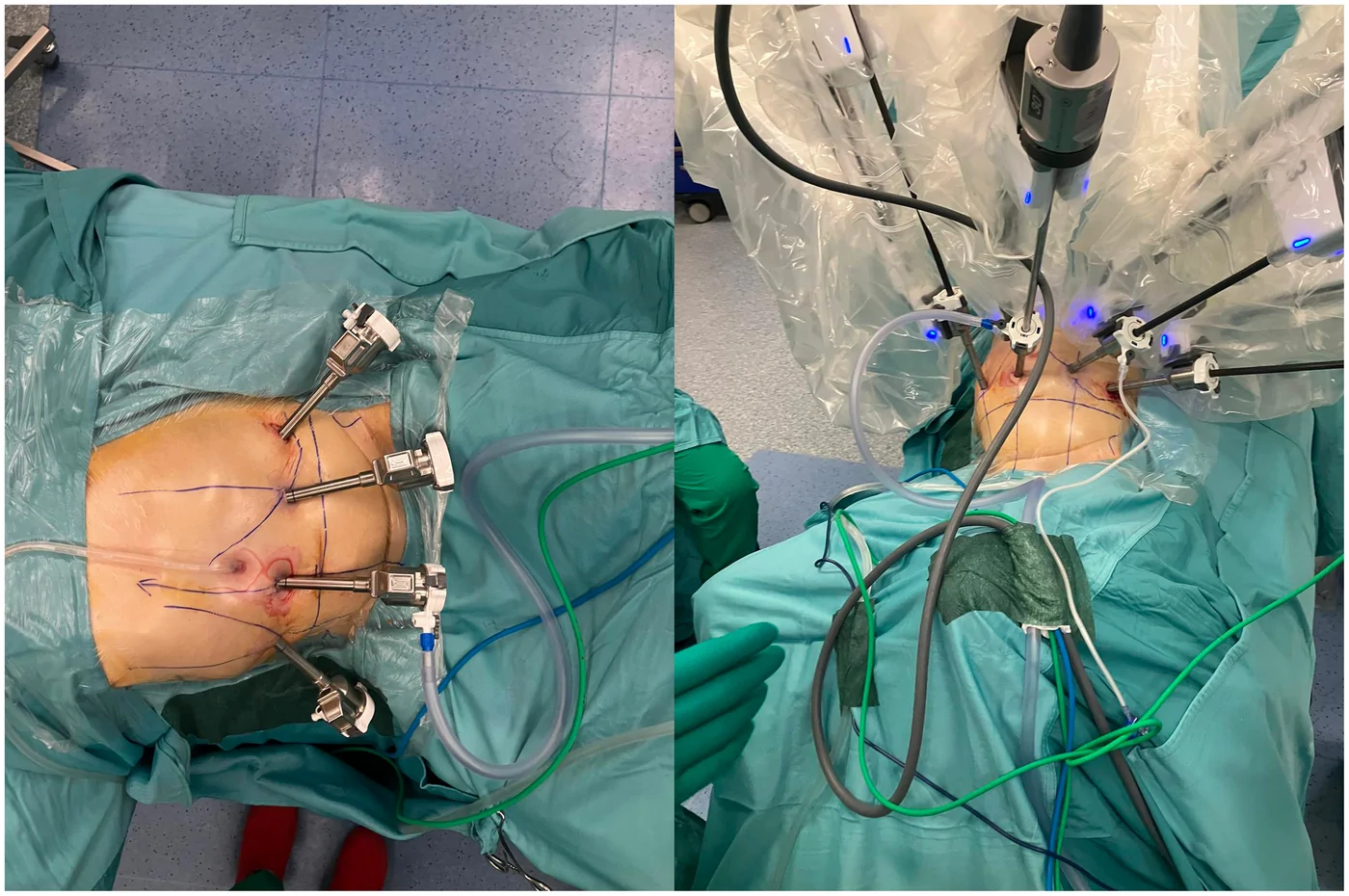
Adapted supine positioning with extensive padding and approximately 20° reverse trendelenburg to accommodate fixed lower-limb contractures while providing access to the upper abdomen.

**Figure 2:**
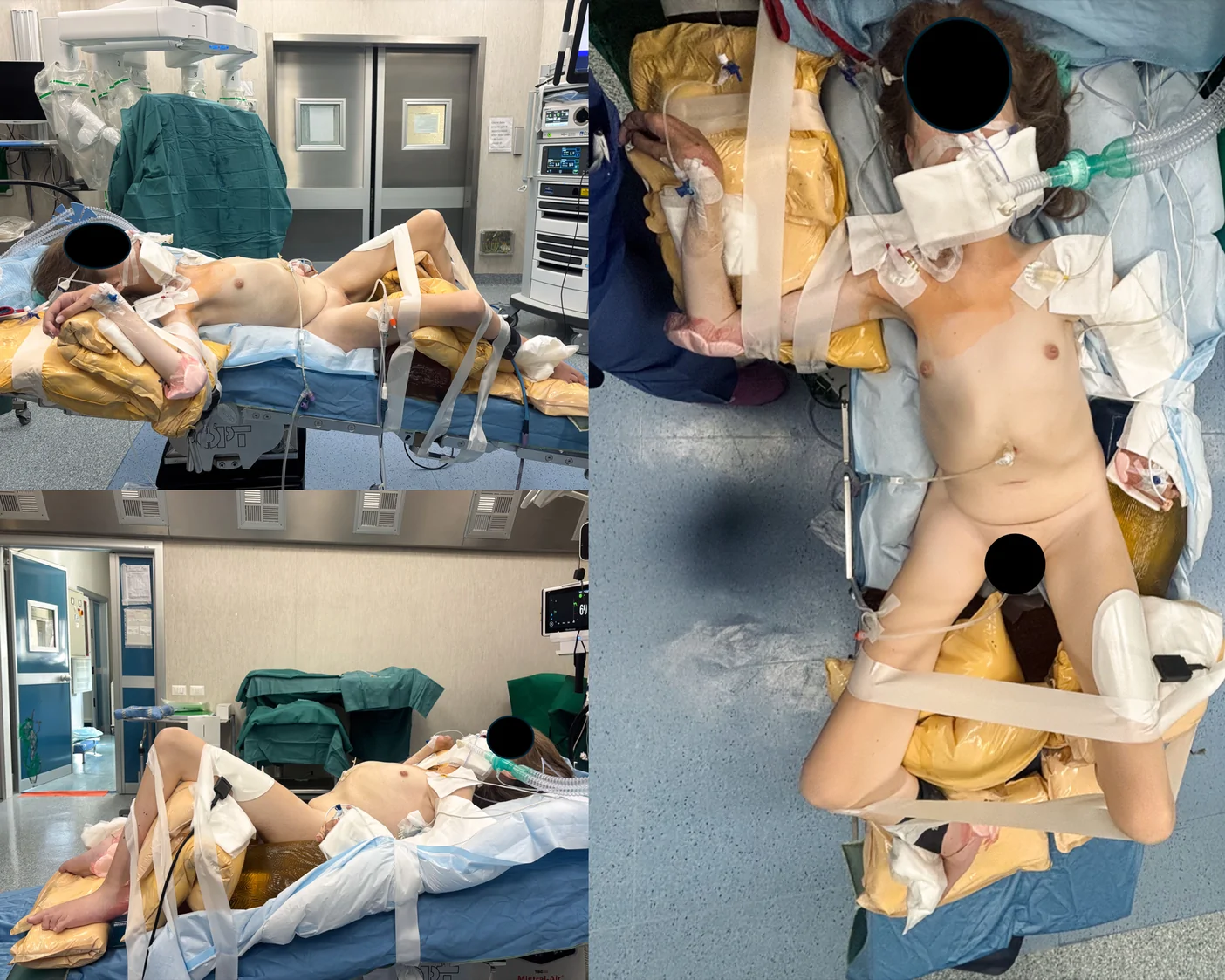
High transverse supraumbilical port placement planned around the pre-existing gastrostomy. The vertical working axis reduced external arm collisions within the restricted abdominal domain.

### Surgical technique

Before docking, upward traction on the trocars was used to “tent” the abdominal wall and expand the restricted working space. Robotic arm 1 was used to elevate the liver, improving visualisation of the oesophageal hiatus without a dedicated liver retractor or an accessory trocar. The instrument sequence included fenestrated bipolar forceps, a needle holder, and monopolar scissors. A 10-Fr nasogastric tube was repurposed as a suction catheter and introduced through one of the robotic ports, providing suction without any additional access. The gastric fundus was mobilised with selective, limited division of short gastric vessels, followed by a 360° Nissen fundoplication. The preoperatively planned Mikulicz pyloroplasty was performed to facilitate gastric emptying and reduce postoperative abdominal distension; this additional procedure modestly prolongs operative time without substantially increasing procedural complexity or anticipated risk (Video 1). Patient positioning required 30 min, trocar placement required 15 min, console time was 65 min, and undocking with abdominal-wall closure required 30 min. Operative time from skin incision to completion of skin closure was therefore 110 min, and total procedural time from the start of positioning to skin closure was 140 min.

### Follow-up and outcomes

The patient was transferred intubated to the ICU. Extubation occurred 2 h 43 min after surgery following complete neuromuscular recovery, wakefulness, an adequate spontaneous breathing pattern, and SpO₂ 100% on an FiO₂ of 21%. She was extubated directly onto prophylactic NIV (inspiratory pressure 16 cmH₂O, expiratory pressure 13 cmH₂O, FiO₂ 21%) in accordance with perioperative recommendations for neuromuscular disease (20). No immediate respiratory event occurred during 24 h of ICU observation. No positioning-related pressure injury was observed postoperatively.

On postoperative day 3, a contrast study demonstrated normal transit without recurrent reflux or leakage from the pyloroplasty. Enteral nutrition through the pre-existing gastrostomy was resumed and progressively increased with good tolerance. No in-hospital complications occurred, and the patient was discharged home on postoperative day 5. At 1 month, a control contrast study confirmed the absence of reflux and satisfactory gastric emptying. Because difficulty managing salivary secretions persisted, although markedly reduced compared with the preoperative period, a further contrast study was performed at 3 months and again demonstrated no gastroesophageal reflux. At the 4-month multidisciplinary assessment, the patient reported that ongoing logopedic therapy, combined with the absence of reflux as an aggravating factor, had improved her ability to manage salivary pooling in the valleculae and enhanced her quality of life with respect to dysphagia-related symptoms. These outcomes were patient-reported and were not assessed using a validated quality-of-life instrument.

## Discussion

Published experience supports minimally invasive fundoplication in selected neurologically impaired patients, although recurrence and reoperation remain more frequent than in neurologically normal populations (21, 22). Disease-specific evidence in SMA is limited to laparoscopy. Yuan et al. reported seven patients with type I or severe type II disease undergoing gastrostomy and Nissen fundoplication with NIV-supported early extubation, while Yang et al. described a child with SMA2 managed with careful neuromuscular and postoperative respiratory strategies (8, 9). Compared with these reports, our patient presented extreme airway risk and major positional constraints. The robotic experience of Margaron et al. in 15 neurologically impaired children with pre-existing gastrostomy provided a relevant technical precedent (10); to our knowledge, the present case is the first to combine these robotic and disease-specific pathways in SMA.

From an anaesthetic perspective, our strategy was driven by the very small margin for airway failure or apnoea. Graham et al. reported low but clinically relevant intraoperative and postoperative complication rates across 56 anaesthetics in children with SMA, many of whom were already dependent on ventilatory support (23), and Islander emphasized the risks of succinylcholine and possible sensitivity to non-depolarising agents (17). In our patient, a mouth opening below 5 mm and a fixed head position made conventional laryngoscopy impracticable, while severe restrictive impairment made repeated attempts after induction unacceptable. We therefore selected nasotracheal fibreoptic intubation under deep sevoflurane sedation and topical anaesthesia, maintaining spontaneous ventilation until the airway had been secured. This differed from the strategy adopted by Chen et al., who avoided neuromuscular blockade during scoliosis surgery, but was consistent with Yang et al., who used monitored non-depolarising blockade for laparoscopic fundoplication (9, 18). In our case, rocuronium was administered only after successful intubation and was then titrated to provide the immobility required for robotic surgery. Planned early extubation directly onto NIV linked the intraoperative strategy to postoperative respiratory support, as in previous SMA experiences (8, 9, 20).

From a surgical perspective, our centre's experience in the robotic treatment of complex neurologically impaired patients had enabled the surgical and anaesthetic teams to develop a coordinated workflow. The robotic approach was not selected on the assumption that it is generally superior to laparoscopy. In this patient, however, the fixed position of the lower limbs and torso restricted access to the upper abdomen, and the articulated instruments provided a practical ergonomic and technical solution within the limited working space. This allowed the procedure to be completed efficiently, with a console time of 65 min, without intraoperative technical difficulties or the need for additional access.

The suitability of the robotic approach also depends on operative duration. Albassam et al. found comparable operative times for robotic and laparoscopic Nissen fundoplication (24), whereas Binet et al. showed that mean operative time fell to 80 ± 10 min after the first 20 robotic cases (25). Salö et al. similarly reported a reduction in fundoplication time from 254 to 181 min as their robotic programme matured (26). Our console time of 65 min, operative time of 110 min, and total procedural time of 140 min including positioning reflect an established multidisciplinary robotic workflow. In a patient with a forced vital capacity of 25% predicted, limiting operative time is a component of safety rather than merely efficiency, because prolonged pneumoperitoneum may worsen respiratory mechanics and carbon dioxide elimination (19). If the robotic learning curve were expected to prolong surgery substantially, the advantages of minimally invasive surgery could be lost and the anaesthetic burden become unacceptable.

### Limitations

Four months of follow-up is insufficient to assess long-term durability, dysphagia, gas-bloat symptoms, aspiration events, or wrap-related complications. Clinical improvement was patient-reported and was not evaluated using a validated quality-of-life instrument.

### Patient perspective

Before surgery, the patient reported severe dysphagia and considerable difficulty managing salivary secretions, with a substantial impact on her daily life and feeding tolerance. She actively participated in the multidisciplinary decision-making process and agreed to undergo surgery after the expected benefits and anaesthetic risks had been discussed. The adult patient had full decision-making capacity and provided written informed consent for publication of the case and the accompanying images and video, which is available for review by the Editor of the journal.

During follow-up, she continued to experience some difficulty managing salivary secretions because of secretion pooling in the valleculae related to her underlying neuromuscular disease; however, these symptoms were markedly reduced compared with the preoperative period. A contrast study performed 3 months after surgery confirmed the absence of gastroesophageal reflux. At 4 months, the patient reported an overall improvement, including better tolerance of enteral nutrition and, with ongoing logopedic therapy aimed at improving vallecular clearance, more effective management of pooled secretions.

## Conclusion

Robot-assisted Nissen fundoplication was feasible despite the combined anaesthetic and surgical complexity of SMA2. At the 4-month follow-up, the patient reported a clinical improvement in symptoms previously aggravated by gastroesophageal reflux. This experience highlights the value of individualized airway planning and coordinated multidisciplinary expertise within an established robotic programme.

## Data Availability

The raw data supporting the conclusions of this article will be made available by the authors, without undue reservation.

## Supplementary material

Supplementary Video S1 Key operative steps of robot-assisted Nissen fundoplication and Mikulicz pyloroplasty, including abdominal-wall tenting, liver elevation using robotic arm 1, and suction through a 10-Fr nasogastric tube introduced via a robotic port.
