# Supplementary material for: Case Report: Robot-assisted nissen fundoplication in spinal muscular atrophy type 2—a tailored surgical and anaesthetic strategy
Source: Front Surg. 2026 Sep 21;13:1964655. doi: 10.3389/fsurg.2026.1964655 (PMC13635347; doi:10.3389/fsurg.2026.1964655)
Supplement: Supplementary file 1 [file Table1.docx]

| **Time point** | **Clinical event** |
| --- | --- |
| **6–8 months of age** | Started crawling, although with some difficulty. |
| **Approximately 1 year of age** | Achieved standing only with support; independent ambulation was not acquired. |
| **July 2003** | Molecular diagnosis of spinal muscular atrophy type 2 (SMA2). |
| **Childhood** | A cough-assist device was introduced during primary-school years. |
| **October 2013** | Spinal fusion for severe scoliosis. |
| **2014** | Initiation of regular nocturnal non-invasive ventilation. |
| **2016** | Initiation of risdiplam within the SUNFISH clinical trial, without adverse effects. |
| **February 2020** | Pneumococcal pneumonia, successfully treated at home. |
| **Summer 2022** | Mild SARS-CoV-2 infection, with symptoms resolving after approximately 5 days. |
| **February–March 2023** | Progressive dysphagia, transition from solid to blended food, and approximately 10-kg weight loss. |
| **May 2024** | Radiologically inserted gastrostomy for severe malnutrition. |
| **July 2024** | Planned otorhinolaryngological procedure abandoned after failed direct and video-laryngoscopic intubation. |
| **Preoperative assessment** | Upper gastrointestinal contrast study demonstrated severe gastroesophageal reflux in the context of impaired secretion management. |
| **April 2026** | Robot-assisted Nissen fundoplication |

**Table 1**
