# Supplementary material for: Case Report: Robot-assisted nissen fundoplication in spinal muscular atrophy type 2—a tailored surgical and anaesthetic strategy
Source: Front Surg. 2026 Sep 21;13:1964655. doi: 10.3389/fsurg.2026.1964655 (PMC13635347; doi:10.3389/fsurg.2026.1964655)
Supplement: Supplementary file 2 [file Table2.docx]

**Legends**

**Table 1.** Clinical timeline summarizing the patient’s disease course

**Figure 1.** Adapted supine positioning with extensive padding and approximately 20° reverse Trendelenburg to accommodate fixed lower-limb contractures while providing access to the upper abdomen.

**Figure 2.** High transverse supraumbilical port placement planned around the pre-existing gastrostomy. The vertical working axis reduced external arm collisions within the restricted abdominal domain.

**Video 1.** Key operative steps of robot-assisted Nissen fundoplication and Mikulicz pyloroplasty, including abdominal-wall tenting, liver elevation using robotic arm 1, and suction through a 10-Fr nasogastric tube introduced via a robotic port.
